# Immunophenotypic characterization of TCR γδ T cells and MAIT cells in HIV-infected individuals developing Hodgkin’s lymphoma

**DOI:** 10.1186/s13027-021-00365-4

**Published:** 2021-04-17

**Authors:** Christina K. S. Muller, Julian Spagnuolo, Annette Audigé, Andrew Chancellor, Doris Russenberger, Alexandra U. Scherrer, Matthias Hoffmann, Roger Kouyos, Manuel Battegay, Gennaro De Libero, Roberto F. Speck, the Swiss HIV Cohort Study, K. Aebi-Popp, A. Anagnostopoulos, M. Battegay, E. Bernasconi, J. Böni, D. L. Braun, H. C. Bucher, A. Calmy, M. Cavassini, A. Ciuffi, G. Dollenmaier, M. Egger, L. Elzi, J. Fehr, J. Fellay, H. Furrer, C. A. Fux, H. F. Günthard, D. Haerry, B. Hasse, H. H. Hirsch, M. Hoffmann, I. Hösli, M. Huber, C. R. Kahlert, L. Kaiser, O. Keiser, T. Klimkait, R. D. Kouyos, H. Kovari, B. Ledergerber, G. Martinetti, B. Martinez de Tejada, C. Marzolini, K. J. Metzner, N. Müller, D. Nicca, P. Paioni, G. Pantaleo, M. Perreau, A. Rauch, C. Rudin, A. U. Scherrer, P. Schmid, R. Speck, M. Stöckle, P. Tarr, A. Trkola, P. Vernazza, G. Wandeler, R. Weber, S. Yerly

**Affiliations:** 1 Department of Infectious Diseases and Hospital Epidemiology, University Hospital of Zurich, University of Zurich, Zurich, Switzerland; 2 Department of Infectious Diseases and Hospital Hygiene, University Hospital of Basel, Basel, Switzerland; 3 Institute of Medical Virology, University of Zurich, Zurich, Switzerland; 4 Division of Infectious Diseases and Infection Control, Cantonal Hospital, St. Gallen, Switzerland

**Keywords:** HIV, Hodgkin’s lymphoma, MAIT cells, T-cell receptor (TCR) γδ cells

## Abstract

**Background:**

Despite successful combined antiretroviral therapy (cART), the risk of non-AIDS defining cancers (NADCs) remains higher for HIV-infected individuals than the general population. The reason for this increase is highly disputed. Here, we hypothesized that T-cell receptor (TCR) γδ cells and/or mucosal-associated invariant T (MAIT) cells might be associated with the increased risk of NADCs. γδ T cells and MAIT cells both serve as a link between the adaptive and the innate immune system, and also to exert direct anti-viral and anti-tumor activity.

**Methods:**

We performed a longitudinal phenotypic characterization of TCR γδ cells and MAIT cells in HIV-infected individuals developing Hodgkin’s lymphoma (HL), the most common type of NADCs. Cryopreserved PBMCs of HIV-infected individuals developing HL, matched HIV-infected controls without (w/o) HL and healthy controls were used for immunophenotyping by polychromatic flow cytometry, including markers for activation, exhaustion and chemokine receptors.

**Results:**

We identified significant differences in the CD4^+^ T cell count between HIV-infected individuals developing HL and HIV-infected matched controls within 1 year before cancer diagnosis. We observed substantial differences in the cellular phenotype mainly between healthy controls and HIV infection irrespective of HL. A number of markers tended to be different in Vδ1 and MAIT cells in HIV^+^HL^+^ patients vs. HIV^+^ w/o HL patients; notably, we observed significant differences for the expression of CCR5, CCR6 and CD16 between these two groups of HIV^+^ patients.

**Conclusion:**

TCR Vδ1 and MAIT cells in HIV-infected individuals developing HL show subtle phenotypical differences as compared to the ones in HIV-infected controls, which may go along with functional impairment and thereby may be less efficient in detecting and eliminating malignant cells. Further, our results support the potential of longitudinal CD4^+^ T cell count analysis for the identification of patients at higher risk to develop HL.

**Supplementary Information:**

The online version contains supplementary material available at 10.1186/s13027-021-00365-4.

## Introduction

Combined antiretroviral therapy (cART) is highly efficient in suppressing HIV replication with life expectancies of HIV-infected individuals nowadays being almost similar to the general population [1–4]. In particular, a significant decline of AIDS-defining cancers (ADCs), such as Kaposi Sarcoma and non-Hodgkin’s lymphoma, has been observed upon cART [5–7]. Strikingly, the percentage of cancer-induced death increased from approximately 10% in the pre-cART era to 28% in the era of cART [8, 9]. The increased cancer burden observed is associated with a higher risk of HIV-infected individuals to develop non-AIDS defining cancers (NADCs) including lung cancer, colorectal cancer, hepatocellular carcinoma, anal cancer, and Hodgkin’s lymphoma (HL) [10–14]. The overall risk of those cancers is three-fold higher for HIV-infected individuals than for the general population and even 5 to 30-fold higher for HL, the most common type of NADCs [10, 15–17].

NADCs are associated with many factors including antiretroviral drug toxicity, aging, and known risk factors such as alcohol and tobacco smoking, but they do not fully explain the higher risk of HIV-infected people to suffer from NADCs [18–20]. Moreover, chronic immune activation, persistent immunodeficiency, as well as co-infections seem to contribute to this increased risk [10, 21–23]. In HIV-infected individuals, for example, almost all cases of HL are associated with EBV infection versus 40% in the general population [24]. In addition, they are also more likely to develop mixed cellular and lymphocyte-depleted subtypes of HL, which are associated with a less favorable outcome than the nodular sclerosis subtype, which is predominant in the general population [25, 26]. The higher incidence rate and development of subtypes with less favorable outcome might be linked to HIV-associated immune dysfunction such as the depletion and/or functional impairment of cells involved in immune surveillance against cancer [24, 27]. Even though the age-specific rates are projected to decrease through 2030 for a number of tumor types, most likely due to the timely treatment of HIV, we are currently challenged with a still increased incidence rate of HL [28]. In fact, in a recent study by Cornejo-Juarez et al., HL figured as the most frequent NADCs in an oncology unit [29].

Strong associations of low CD4^+^ T cell counts and ADCs are well proven, while associations of CD4^+^ T cell counts and NADCs are weaker or not observed for all NADCs [17, 19, 23, 30–32]. Further, the immediate initiation of cART reduces serious AIDS-related and non-AIDS related events [33, 34]. The START study, for example, showed a reduced risk for cancers (ADCs and NADCs) when cART was initiated immediately and irrespective of CD4^+^ T cell count but there was no evidence that this beneficial effect was associated with CD4^+^ T cell count or viral load [34, 35]. Thus, the higher risk for NADCs is not simply an equation of CD4^+^ T cell counts but likely involves more complex mechanisms of immune activation and immune surveillance.

TCR γδ cells and mucosal-associated invariant T (MAIT) cells are innate-like T lymphocytes with important functions in both innate and adaptive immune response [36–39]. TCR γδ cells express an invariant T cell receptor (TCR), composed of a gamma (γ) and a delta (δ) chain. They usually comprise 0.5–16% of all CD3^+^ T cells in the peripheral blood (PB) but can expand to up to 60% during bacterial and viral infections [40–42]. Based on their TCR Vδ chain usage, they can be divided into two major sub-populations; namely, Vδ1- and Vδ2-expressing cells. A minority of TCR γδ cells instead express Vδ chains other than these two. Tissue-associated TCR γδ T cells mostly express the TCR Vδ1 chain, whereas cells expressing the Vδ2 chain (usually paired with the Vγ9 chain, and therefore also called Vγ9Vδ2 cells) are the predominant population in the PB [43]. Upon HIV infection, an expansion of Vδ1 cells accompanied by a depletion of Vδ2 cells in the PB is observed, resulting in an inversion of the Vδ1/ Vδ2 ratio [44–47]. TCR γδ cells perform diverse functions and are also involved in anti-viral and anti-tumor activity [48]. They can rapidly secrete large amounts of IFN-γ, TNF-α, IL-4 and IL-17, and can act as potent cytotoxic effector cells against virus-infected and malignant cells through the release of perforin and granzyme-B [49–52]. These cells may also have a regulatory function via secretion of IL-10 and TGF-β with potential suppressive effects on anti-tumor function [53]. The role of TCR γδ cells in tumor immunity is not fully understood, and further analysis of individual populations based on the TCR-repertoire and functional heterogeneity is needed [54].

MAIT cells, which are abundant in the PB, mesenteric lymph nodes, liver and intestinal mucosa, are also involved in tumor immunity [55–58]. They express the semi-invariant TCR Vα7.2, paired with a limited Vβ repertoire and the C-type lectin CD161 [59, 60]. Similar to TCR γδ cells, MAIT cells can rapidly secrete cytokines, including IFN-γ, TNF-α, IL-17, and IL-22, and may also kill target cells [55, 59, 61, 62]. Early during HIV infection, MAIT cells are depleted and functionally impaired and like TCR γδ cells, do not fully recover upon long-term cART [63–65].

As MAIT and TCR γδ cells are involved in antitumor immunity, we hypothesized that the extent of their depletion and/or their phenotype differ between HIV-infected patients developing HL (prior to the diagnosis of HL) and HIV-infected matched controls. Notably, extensive characterization of immune cells in the PB of HIV-infected individuals over time may provide detailed insight on their immune reconstitution and on the phenotype of cellular populations with distinct functions, and could also provide useful predictors of disease progression. To address this possibility, we performed a detailed phenotypic characterization of TCR γδ and MAIT cells in the PB of HIV-infected individuals enrolled in the Swiss HIV Cohort Study (SHCS).

## Material and methods

### Swiss HIV cohort study (SHCS)

The SHCS (www.shcs.ch) is a prospective cohort study with ongoing enrollment of HIV-infected adults in Switzerland since 1988 [66]. It includes 73% of all diagnosed HIV-infections in Switzerland [67]. Representation has remained stable throughout the study duration. Detailed information on demographics, mode of HIV acquisition, risk behavior, clinical events, co-infections, and treatment is collected using a standard protocol at registration and at intervals of 6 months. Plasma samples are collected every 6–12 months in all study participants. Local ethical committees of all participating study sites approved the study and written consent was obtained from all participants.

### Samples

Blood was obtained from healthy controls and HIV-infected patients with and without HL, which were enrolled in the Swiss HIV Cohort Study (SHCS). Peripheral blood mononuclear cells (PBMCs) were isolated using Lymphoprep gradients and cryopreserved. Within the SHCS demographic, clinical, laboratory and behavioral data are recorded at enrolment and at follow-up visits every 6 months. Samples were selected based on the following inclusion criteria: i) male, ii) ≥ 18 years, iii) Caucasian, iv) HIV RNA copies > 400 copies/ml, v) sample availability before cART, 1–2 years after suppression, and 0–1 years prior to HL diagnosis, i.e.*,* for the HIV-patients w/o HL we chose the samples closest to the times of the corresponding matching HIV^+^ HL^+^ patients. Matching of cancer-free HIV-infected individual was done according to: i) gender, ii) ethnicity, iii) age, iv) sample availability, v) CD4^+^ T cell count (before cART), vi) HIV RNA copy number (before cART).

### Flow Cytometry

Frequencies and cell count of conventional CD4^+^ and CD8^+^ T cells were determined throughout the study and provided by the SHCS, frequencies of unconventional γδ T cells and MAIT cells were determined retrospectively. Samples were analyzed on two consecutive days. To ensure comparability of the samples, all time points and matched control samples were stained and acquired on the same day. We checked for technical performance by analyzing one healthy control sample on both days. Cryopreserved PBMCs were thawed, washed, and resuspended in phosphate buffered saline (PBS). Cell number after thawing was determined with the COULTER® Ac · T diff™ Analyzer (Beckman Coulter). Three different polychromatic flow cytometry panels were used for the identification and characterization of γδ T cells and MAIT cells. Each staining step included incubation for 20 min at 4 °C. One million PBMCs were used per panel and stained with purified anti-TCRγδ (BD Bioscience) and the Zombie NIR Fixable Viability dye (BioLegend) in PBS with 2 mM. PBMCs were washed 2x and then stained with anti-mouse IgG (H + L) – Pacific Orange (Thermo Fisher Scientific) in FACS buffer (PBS containing 2% FBS and 0.05% sodium azide). PBMCs were washed 2x, followed by a 20 min blocking step with mouse serum (Thermo Fisher Scientific) at 4 °C. After blocking, cells were washed and surface staining with three different panels was performed. Each panel included anti-TCRVδ1 - PE-Vio770 (Miltenyi Biotec), anti-TCRVδ2 - PerCP (BioLegend), anti-CD161 - BV711 (BD Bioscience) and anti-TCRVα7.2 – BV785 (BioLegend), plus, Panel 1: anti-CCR5 – APC, anti-CCR6 – PE, anti-CXCR3 – PE-Dazzle, anti-CXCR4 – BV421, anti-CD38 – BV605, and anti-CD69 – FITC (all BioLegend); Panel 2: anti-NKG2D –BV605 (BD Bioscience), anti-CD94 – FITC, anti-Tim3 – PE-Dazzle, anti-PD-1 – BV421, anti-ILT2 – PE, anti-CD158b – APC (all BioLegend); and Panel 3: anti-CD16 – FITC (BD Bioscience), anti-KLRG1 – PE, anti-CTLA4 – BV421, anti-CD57 – PE-Dazzle, anti-CD56 – APC (all BioLegend). Before acquisition, cells were fixed with 1% paraformaldehyde. Samples were acquired on a BD LSR II Fortessa (BD Bioscience). Ultra Comp eBeads (Thermo Fisher Scientific) were used for compensation, except for anti-CD57 – PE-Dazzle and the Zombie NIR Fixable Viability dye, for which compensation was done with PBMCs. Anti- TCRVδ1 - PE-Vio770 was compensated using the MACS Comp bead Kit, anti REA (Miltenyi Biotec). Data were analyzed using FlowJo software (TreeStar). All results shown included gating on lymphocytes, single cells, and live cells. Detailed subset analysis of γδ T cells was performed by gating on TCRγδ^+^/TCRVδ1^+^ or on TCRγδ^+^/TCRVδ2^+^ cells. MAIT cells gated based on TCRVα7.2^+^/CD161^+^. Subset analysis was only performed when a threshold of 100 detected events for the parental population was reached.

### Quantification and statistical analysis

Results on frequencies of δγ T cells, Vδ1, Vδ2 cells, and MAIT cells were extracted from all three panels. The mean frequency was calculated and used for further analysis. Data was only plotted when results were available for four or more patients per group. Statistical data analyses were performed using GraphPad Prism 8 software (GraphPad). The ROUT method was used for the identification of outliers. Data were subjected to a Wilcoxon signed-rank test or Kruskal-Wallis with Dunn’s multiple comparison test. *P* values were considered as significant at *p* < 0.05.

All analysis was performed within the R statistical computing environment, version 3.6.3 [68]. FACS data from the three panels (CCR, Exhaustion and NK) and population (MAIT, Gamma-delta VdX, Vd1 and Vd2) were analyzed independently, using a common analytical framework described here. Data was first zero-centered on gate-values for each marker and arcsin transformed before dimensional reduction and clustering using Rphenograph [69]. The proportions of HC, HIV^+^ w/o HL and HIV^+^ HL^+^ cells and median marker expression within each cluster were calculated and heatmapped to visualize phenotypic signatures. To train the SVM classifier, a training set containing equal numbers of cells from each of the three conditions was obtained by sampling using the caret package [70]. Training data for each population (MAIT, Gamma-delta VdX, Vd1 and Vd2) were used to build separate SVM model using the e1071 package [71] under default parameters. These models were then used to predict condition (HC, HIV^+^ w/o HL or HIV^+^ HL^+^) for each cell in the dataset with probabilities for each condition. The median predicted condition probability was calculated for each phenograph cluster. Similarly, down-sampled datasets were presented to the DDRTree and Slingshot [72, 73] algorithms for pseudotemporal ordering based on phenotypic markers (i.e., non-lineage) alone.

## Results

### Patient characteristics

We screened the entire SHCS cohort for cryopreserved PBMC samples of HIV-infected individuals developing NADCs and identified a group of 10 patients developing HL (HIV^+^ HL^+^) (Table 1). Even though the SHCS enrolls more than 70% of all HIV-infected individuals in Switzerland [67], the number of patients with other NADCs and sufficient sample availability was 2 to 3 and thus did not justify their inclusion in our study. In addition, we included matched HIV-negative healthy controls (HC) in this study (*n* = 10) (Table 1). The identification of the specimens at the various time points was only feasible thanks to the biannually biobanking of specimens from all patients in the SHCS cohort.

**Table 1:** SHCS Patient Characteristics

	HIV+ w/o HL group (***n*** = 10)	HIV+ HL+ group (***n*** = 10)	w/o HL vs. HL+ (***p***-value)
**Gender**			
Male	10	10	NA
**Ethnicity**			
Caucasian	10	10	NA
**Age at HIV diagnosis,** median (IQR)	34.5 (28.75, 38)	31 (26.75, 39)	0.1211
**Baseline plasma viral load** (copies per ml), median (IQR)	25,865 (11,730, 51,475)	24,092 (14,550, 739,500)	0.1602
**Baseline CD4**^**+**^ **T cell count per ul**, median (IQR)	523 (368, 602)	448 (310, 526)	0.3750
**Years between HIV diagnosis and initiation of cART,** median (IQR)	3 (1.5, 3.25)	2 (0, 2.5)	0.4688
**Duration cART in days,** median (IQR)	976 (717, 1457)	1382 (568, 2328)	0.3750
**cART included EFV** (%)	30%	60%	0.3698^i^
**Age at HL diagnosis,** median (IQR)	NA	39 (34, 44.25)	

Abbreviations: *cART* combined antiretroviral therapy, *HL* Hodgkin’s lymphoma, *EFV* Efavirenz

Statistical analysis: Wilcoxon signed-rank test and ^i^exact Fisher’s test

### Dynamics of T cell populations in HIV-infected patients with and without HL

We analyzed CD4^+^ and CD8^+^ T cell counts and frequencies of TCR γδ and MAIT cells in the PB of HIV-infected individuals prior to cART, 1–2 years after the suppression of plasma viremia, 0–1 year before HL diagnosis.

**Fig. 1:**
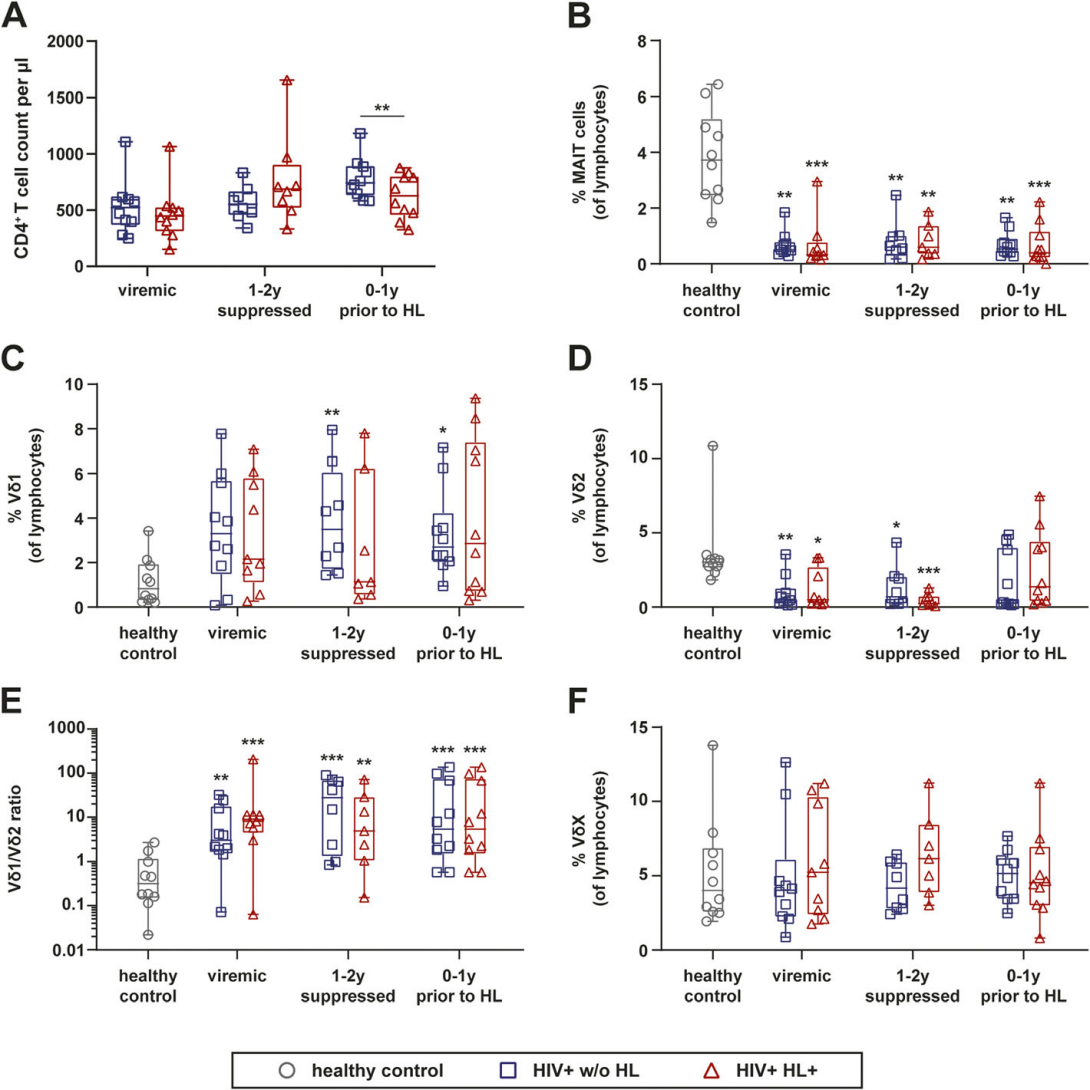
Longitudinal analysis of T cell populations in the PB of HIV-infected individuals with and without HL. **a** CD4^+^ T cell count per μl in the PB of HIV-infected individuals developing HL (HIV^+^ HL^+^) and matched controls (HIV^+^ w/o HL). **b-e** Distribution of γδ T cells and MAIT cell in the PB of HIV-infected individuals developing HL (HIV^+^ HL^+^), matched controls (HIV^+^ w/o HL), and healthy controls. **b** Frequencies of MAIT cells of total lymphocytes. **c** Frequencies of Vδ1 cells of γδ T cells. **d** Frequencies of Vδ2 cells of γδ T cells. **e** Ratio of Vδ1 cells and Vδ2 cells. **f** Frequencies of VδX cells of total lymphocytes. Whiskers represent minimum and maximum. Analysis of HIV^+^ HL^+^ group versus HIV^+^ control group w/o HL by Wilcoxon signed-rank test, comparison of HIV-infected groups versus HC by Kruskal Wallis with Dunnett’s multiple comparison test. * without indicating line represent significance compared to healthy control. **** *p* ≤ 0.0001; *** *p* ≤ 0.001; ** *p* ≤ 0.01; *p ≤ 0.05. (PB) peripheral blood, (HL) Hodgkin’s lymphoma, (MAIT) Mucosal associated invariant T cells

The CD4^+^ T-cell counts were similar between HIV-infected individuals developing HL and their matched HIV-infected controls prior to cART or 1–2 years after successful cART. However, we observed a significantly lower CD4^+^ T cell count in HIV^+^ HL^+^ patients just prior to its diagnosis than in HIV^+^ w/o HL patients (Fig. 1a). The CD8^+^ T-cell counts were similar between the groups at all times assessed (data not shown).

Further, we observed a significant decrease of MAIT cells in HIV-infected patients irrespective of HL, which did not recover following cART (Fig. 1b). TCR γδ T subset analyses revealed a significant expansion of the Vδ1 cell subset in HIV-infected patients w/o HL under cART as compared to HCs (Fig. 1c). At the same time, Vδ2 cells in PB were significantly reduced irrespective of HL (Fig. 1d). This led to an inversion of the Vδ1/ Vδ2 ratio in all HIV-infected patients, which differed significantly from the ratio detected in HCs (Fig. 1e). We did not observe any differences for TCR VδX cells between HIV^+^ HL^+^, HIV^+^ w/o HL and HC at any time point (Fig. 1f). In conclusion, the overall frequencies of γδ T or MAIT cells were similar in HIV-infected patients irrespective of HL diagnosis whereas the CD4^+^ T cell counts were significantly lower in HIV^+^ HL^+^ patients just prior to HL diagnosis as compared to HIV^+^ w/o HL patients.

### HIV infection leads to an increase in activation and exhaustion marker expression

Next, we investigated whether TCR γδ and MAIT cells differ between HIV^+^ HL^+^ patients, their HIV^+^ w/o HL matched controls and HCs in their activation and exhaustion status.

**Fig. 2:**
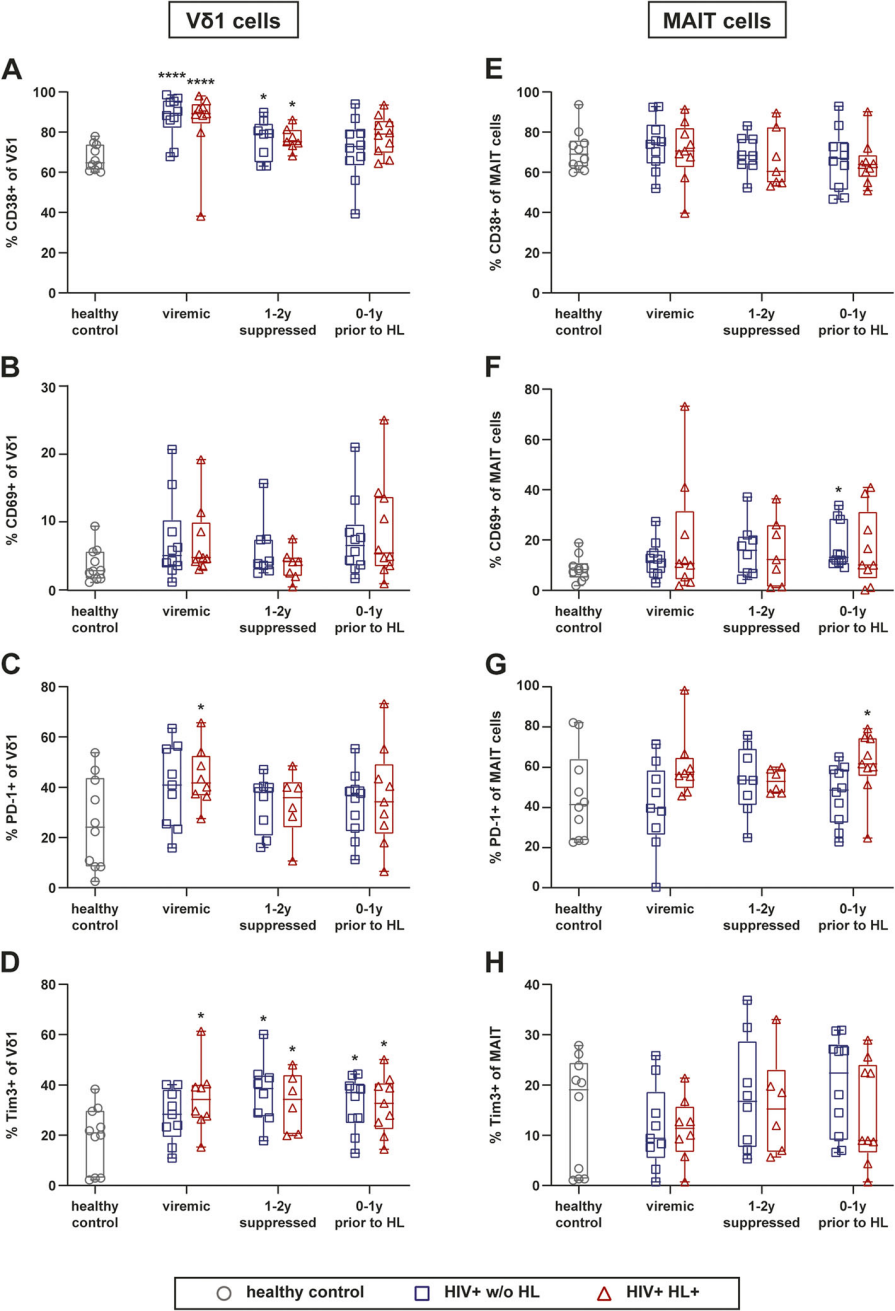
Activation and exhaustion marker analysis on Vδ1 and MAIT cells of HIV-infected individuals with and without HL. **a-d** Frequency of Vδ1 cells positive for activation marker (CD38 and CD69) and exhaustion maker (PD-1 and Tim3) expression. **e-h** Frequency of MAIT cells positive for activation marker (CD38 and CD69) and exhaustion maker (PD-1 and Tim3) expression. Whiskers represent minimum and maximum. Analysis of HIV^+^ HL^+^ group versus HIV^+^ control group w/o HL by Wilcoxon signed-rank test, comparison of HIV-infected groups versus HC by Kruskal Wallis with Dunnett’s multiple comparison test. * without indicating line represent significance compared to healthy control. **** *p* ≤ 0.0001; *** *p* ≤ 0.001; ** *p* ≤ 0.01; *p ≤ 0.05. (PB) peripheral blood, (HL) Hodgkin’s lymphoma, (MAIT) Mucosal associated invariant T cells

We observed main differences between HIV-infected patients irrespective of HL vs. HC. Namely, the frequency of Vδ1 cells expressing the activation marker CD38 increased significantly upon HIV-infection and decreased upon long-term cART (Fig. 2a). This increased frequency of CD38^+^ Vδ1 cells was not observed for MAIT cells (Fig. 2e). Cells expressing the activation marker CD69 showed a similar trend of higher frequencies in HIV infection but was highly variable with solely a significant increased frequency of CD69^+^ MAIT cells in HIV^+^ w/o HL patients 0–1 years prior to HL diagnosis (Fig. 2b and f).

The frequencies of cells expressing the exhaustion marker PD-1 was highly variable across and within the groups at all times assessed with only significant increases of Vδ1 cells in viremic HIV^+^ HL^+^ patients, and of MAIT cells in HIV^+^ HL^+^ patients just prior to HL diagnosis (Fig. 2c and g). The frequencies of Vδ1 cells expressing Tim-3 were overall significantly higher throughout HIV infection when compared to those detected in HCs (Fig. 2d). In MAIT cells, no significant differences in the frequencies of Tim-3^+^ cells were observed (Fig. 2h).

Overall, Vδ1 cells and MAIT cells with an activated and exhausted phenotype were more frequent upon HIV infection, which was only partially reverted upon long-term cART.

### Expression of tissue homing receptors on γδ T cells and MAIT cells is altered upon HIV infection

We also determined the expression of different tissue homing receptors in our cohort, including CXCR3, CCR6, as well as CXCR4 and CCR5, with the latter two serving as viral co-receptors during entry of HIV. Notably, homing is a very critical function for immunosurveillance and its dysfunction a potential indication for insufficient control of infections or tumors.

**Fig. 3:**
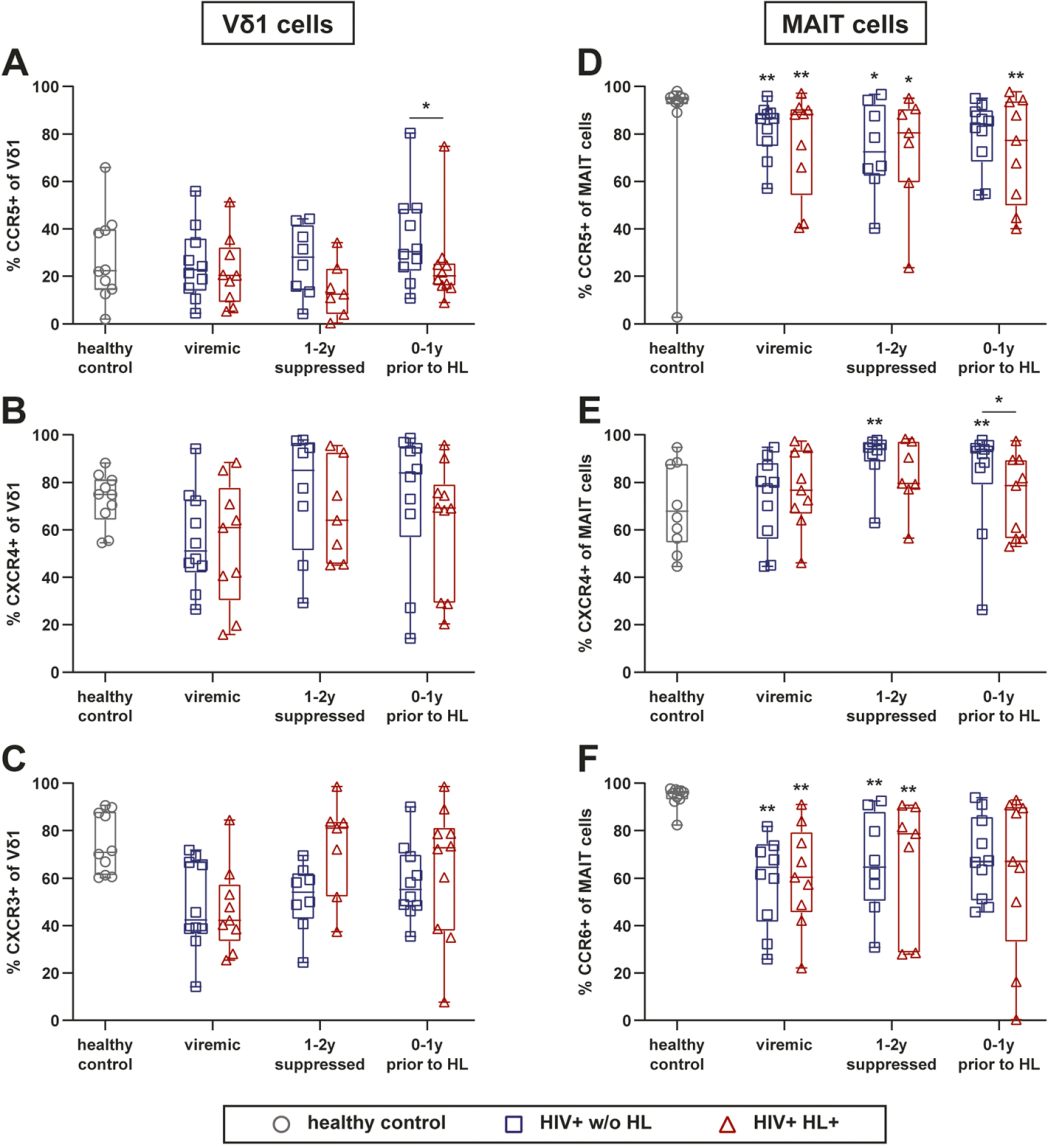
Tissue homing receptor expression on Vδ1 and MAIT cells of HIV-infected individuals with and without HL. **a-c** Frequencies of Vδ1 cells expression CCR5, CXCR3, and CXCR4. **d-f** Frequencies of MAIT cells expression CCR5, CXCR4, and CCR6. Whiskers represent minimum and maximum. Comparison of HIV-infected groups versus HC by Kruskal Wallis with Dunnett’s multiple comparison test. * without indicating line represent significance compared to healthy control. **** *p* ≤ 0.0001; *** *p* ≤ 0.001; ** *p* ≤ 0.01; *p ≤ 0.05. (PB) peripheral blood, (HL) Hodgkin’s lymphoma, (MAIT) Mucosal associated invariant T cells

The frequency of CCR5^+^ Vδ1 cells was significantly higher in HIV^+^ w/o HL vs. HIV^+^ HL^+^ 0–1 years prior to HL diagnosis (Fig. 3a). Instead, a slight but significant decrease in the frequency of CCR5^+^ MAIT cells was observed upon infection with HIV irrespective of HL (Fig. 3d).

Overall, the frequencies of CXCR4^+^ Vδ1 and CXCR4+ MAIT cells were very heterogeneous in HCs and HIV-infected individuals (Fig. 3b and e). Notably, the frequencies of CXCR4^+^ MAIT cells tended to be higher in HIV infection with a significant increase during the viremic phase and 1–2 years after HIV suppression (Fig. 3e). Further, we found a trend towards a decrease of CXCR3^+^ Vδ1 and MAIT cells in HIV infection (Fig. 3c and data not shown). CCR6^+^ Th17-like Vδ1 cells were barely detectable in all groups (data not shown). In MAIT cells, the majority displayed a CCR6^+^ Th17-like phenotype, and the frequency of these cells was significantly reduced upon HIV-infection (Fig. 3f).

### Characterization of natural killer cell markers expression

**Fig. 4:**
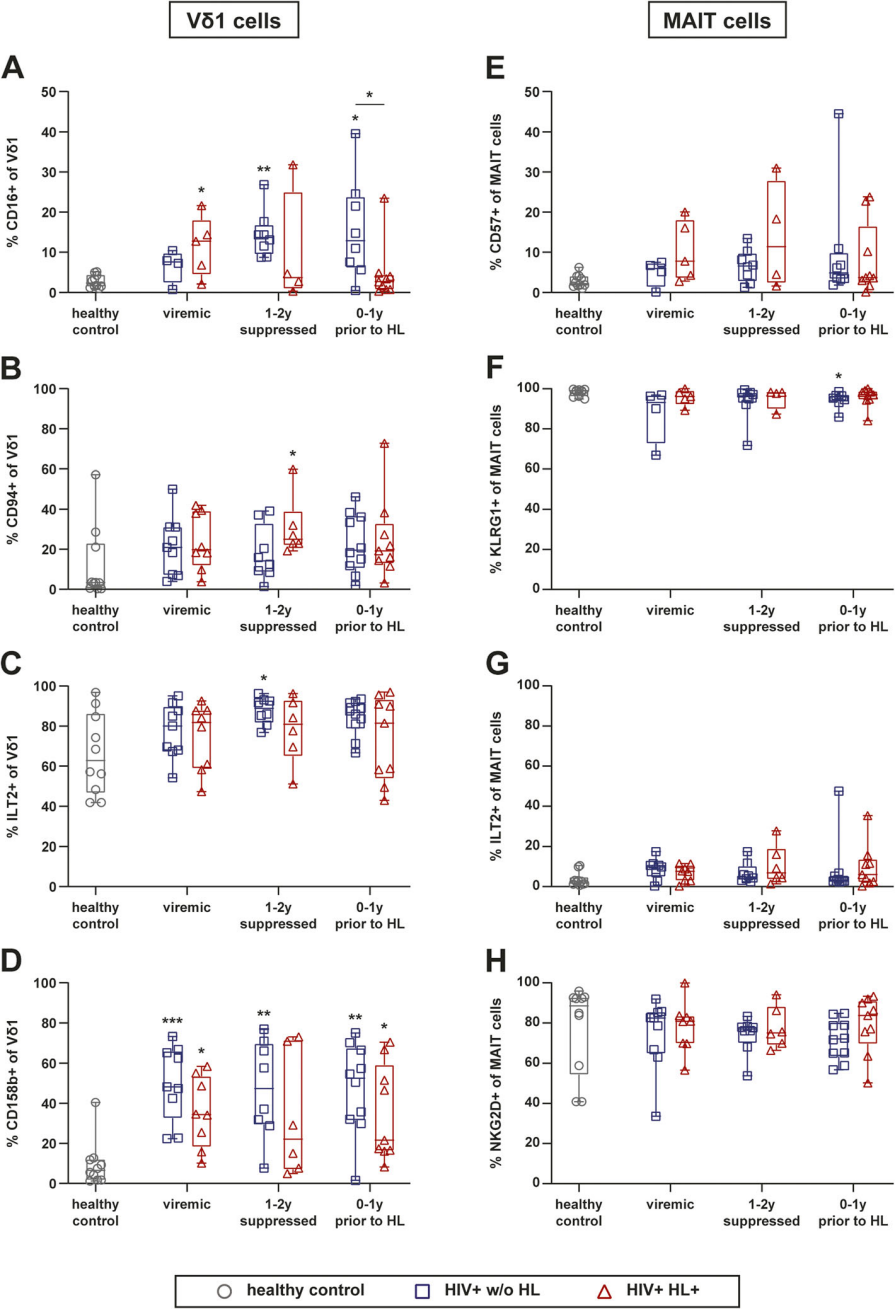
Characterization of natural killer cell marker and receptor expression on Vδ1 and MAIT cells of HIV-infected individuals with and without HL. **a-d** Frequencies of Vδ1 cells being CD16^+^, CD94^+^, CD158b^+^, and ILT2^+^. **e-h** Frequencies of MAIT cells expressing CD57, KLRG1, NKG2D, and ILT2. Whiskers represent minimum and maximum. Analysis of HIV^+^ HL^+^ group versus HIV^+^ control group w/o HL by Wilcoxon signed-rank test, comparison of HIV-infected groups versus HC by Kruskal Wallis with Dunnett’s multiple comparison test. * without indicating line represent significance compared to healthy control. **** *p* ≤ 0.0001; *** *p* ≤ 0.001; ** *p* ≤ 0.01; *p ≤ 0.05. (PB) peripheral blood, (HL) Hodgkin’s lymphoma, (MAIT) Mucosal associated invariant T cells

The phenotype of TCR γδ and MAIT cells was further assessed by investigation of different natural killer (NK) cell-associated receptors. We detected a significantly higher frequency of CD16^+^ Vδ1 and Vδ2 cells in the PB of HIV^+^ w/o HL as compared to HC and to HIV^+^ HL^+^ at various time points (Fig. 4a and Supp. 1E). MAIT cells did not express CD16 (data not shown).

The NK cell-associated receptors CD94 and ILT-2 were expressed by a substantial fraction of Vδ1 cells upon HIV infection (Fig. 4b and c). The percentages of CD94^+^ Vδ1 cells tended to remain elevated despite long-term cART (Fig. 4b). The inhibitory ILT2 receptor was expressed by the majority of TCR Vδ1 cells in HCs and the frequency of these cells tended to increase further upon HIV infection (Fig. 4c). In contrast, only a small fraction of MAIT cells were ILT-2^+^ (Fig. 4g).

The frequencies of Vδ1 cells expressing inhibitory killer cell immunoglobulin-like receptor (KIR) CD158b were significantly increased upon HIV infection. Frequencies of CD158b^+^ Vδ1 cells tended to be higher in HIV^+^ w/o HL individuals than in HIV^+^ HL^+^ patients (Fig. 4d). CD94 and CD158b expression was barely detectable on MAIT cells (data not shown). KLRG1 and NKG2D were expressed by the majority of MAIT cells in HC and HIV^+^ individuals (Fig. 4f and h), but barely detectable on Vδ1 cells.

Taken together, these findings showed a long-term impact of HIV-infection on the frequencies of TCR Vδ1 cells expressing particular NK cell markers, and their frequencies remained even under successful cART.

### Analyses of Vδ2 and the VδX cells

Notably, we also extracted the data for the TCR Vδ2 and TCR VδX cells from our flow cytometric analyses (Suppl. Figures 1 and 2). The overall number of TCR Vδ2 cells were rather limited, and the expression pattern of TCR VδX did reveal only for a few significant differences, thus we only present a subset of the data. Similar to the data in TCR Vδ1 cells, we observed a higher frequency of TCR Vδ2 cells with an activated phenotype and with a decrease in the homing molecule CXCR3 in HIV infection (Suppl. Figure 1A and B). Notably, the TCR Vδ2 cells in patients w/o HL presented a higher frequency of CD16^+^ and KLRG1^+^ cells as compared to HIV^+^ HL^+^ patients. In synopsis, HIV^+^ w/o HL have more terminally differentiated cells (Suppl. Figure 1F and G) and cells expressing CD16 (Suppl. Figure 1E) than HIV^+^ HL^+^ patients. We also observed similarities between TCR Vδ1 and VδX cells. Namely, the higher frequency of CCR5^+^ cells in HIV^+^ w/o HL patients 1–2 years suppression and prior to diagnosis and the increased frequencies of CD158b^+^ cells in viremic HIV^+^ w/o HL patients (Suppl. Figure 2A and D). Further, HIV-infected patients irrespective of HL presented lower frequencies of CCR6^+^ VδX at all times assessed (Suppl. Figure 2B) and CXCR4^+^ VδX cells during the viremic state compared to healthy controls (Suppl. Figure 2C). Apart from those findings, the cell frequencies did not differ significantly between the three groups analyzed.

### Clustering and phenotypic signature analysis of FACS panels

To identify discrete phenotypes within MAIT, TCR Vδx, Vδ1 or Vδ2 populations that distinguish or predict HL within HIV^+^ patients, we used PhenoGraph [74] for visualization and clustering of high-dimensional FACS data. We sought to identify clusters containing a high majority of cells from HIV^+^ HL^+^ patients, indicating a population of cells specific to HIV^+^ HL^+^ vs. HIV^+^ w/o HL patients or HC. In general, the clustering analysis indicated that MAIT and TCR Vδx, Vδ1 or Vδ2 populations in both HIV^+^ HL^+^ and HIV^+^ w/o HL patients shared phenotypes and clustering is most likely driven by differences caused by HIV infection and not HL (Suppl. Figures 3, 4, 5).

Given the complexity of the clustering and difficulty in identifying populations with phenotypic signatures unique to HIV^+^ HL^+^ patients, we trained support vector machine (SVM) classifier models to predict whether a cell comes from HC, HIV^+^ HL^+^ or HIV^+^ w/o HL. Ideally, the probability distributions for the SVM’s prediction should show discrete peaks of high-probability for each of the three groups (HIV^+^ HL^+^, HIV^+^ w/o HL and HC). Instead, we observed that in each population the probability curves for both HIV^+^ HL^+^ and HIV^+^ w/o HL predictions overlapped and the models yielded relatively poor predictions overall, suggesting that phenotypes in both groups were too similar to be distinguished (Suppl. Figures 6, 7, 8) and no phenotypic signatures could be attributed to HIV infection with or without HL.

**Fig. 5:**
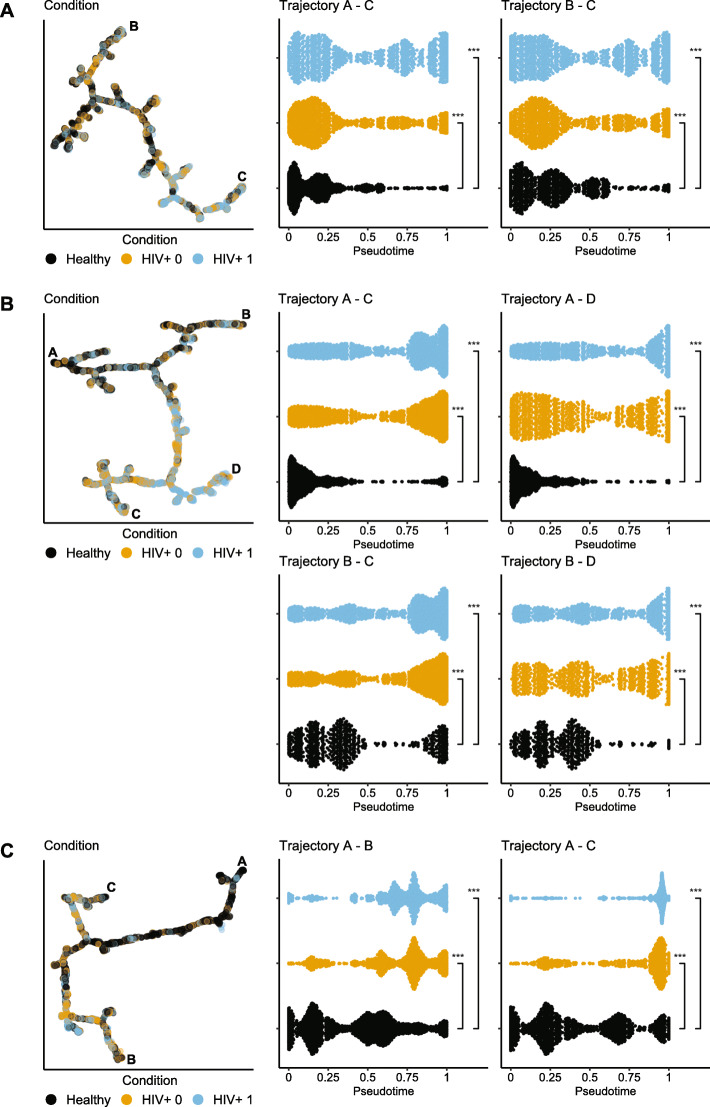
Pseudotemporal ordering of MAIT, Vδ1 and Vδ2 populations. Data for each population, down-sampled evenly over patients, conditions and time-points to 1e4 cells, was used to predict phenotype trajectories using DDRTree and Slingshot. Predicted trajectories, labelled within each tree, show imbalanced distributions of cells from HC, HIV and HIV HL patients in response to CCR expression in MAIT cells (**A**), exhaustion marker expression in Vδ1 (**B**) and NK marker expression in Vδ2 cells (**C**). Asterisks indicate significance of permutation tests (*** *p < 0.001*)

Since the data contained two time-variables (duration of infection and duration of treatment), we next attempted to establish whether there was any time-dependent effect on phenotypes. Therefore, we performed pseudotime analysis using DDRTree trained on the expression data [73, 75](Suppl. Figures 9, 10, 11). If any time-dependent effects on the phenotype were present, a gradient in infection-duration or treatment-duration in relation to the predicted trajectories would have been clearly observed. However, in agreement with the clustering and SVM classifier, cells from HIV^+^ HL^+^ and HIV^+^ w/o HL patients and HC were generally evenly distributed across the tree, indicating that the phenotype of these cell populations in these panels was independent of disease condition. Nevertheless, three populations in separate FACS panels appear to have pseudotemporal-dependent distributions in the DDR Trees, namely MAIT cells in the CCR panel (Fig. 5A), TCR Vδ1 cells in the exhaustion panel, and TCR Vδ2 cells in the NK panel (Fig. 5B and C). However, there was no correlation between pseudotime and duration of viral suppression or disease, indicating that phenotype and therefore, pseudotemporal ordering is driven by HIV infection and not Hodgkin’s lymphoma as indicated by the violin plots.

## Discussion

In the present study, we investigated whether TCR γδ and MAIT cells differ in HIV-infected individuals developing HL from HIV-infected individuals, which otherwise are healthy. The main findings were: i) an association of a lower CD4^+^ T cell count and HL risk 0–1 year before HL diagnosis, and ii) prominent phenotypic changes between HIV infected patients irrespective of HL vs. HC. We observed subtle phenotypic changes in TCR Vδ1 and MAIT cells potentially going along with a greater functional impairment in HIV-infected individuals developing HL. However, clustering and SVM analyses of the data did not identify the presence/loss of unique cell populations associated with HL development. In fact the DDRtree algorithm also supported that the phenotypes observed was driven by HIV infection and not by HL.

Even though we screened the entire SHCS cohort, we only identified a limited number of HIV-infected patients developing HL. For each HIV^+^ HL^+^ patient, we had a matched control.

The CD4^+^ T-cell count, prior to cART, and its initial increase in response to cART were similar in both HIV-infected patient groups. In contrast, the CD4^+^ T cell counts 0–1 year prior to HL diagnosis were lower in patients with HL. This is in line with studies by the French Hospital Database on HIV and Collaboration of Observational HIV Epidemiological Research in Europe (COHERE), strengthening the potential of the CD4^+^ T cell count as a surrogate marker to identify HIV^+^ patients with a high risk to develop HL [17, 76–78]. The decline in the CD4^+^ T cell count might be explained by the sequestration of lymphocytes to the growing tumor, or by a lack of continuing T-cell recovery, which might result in a lack of EBV-specific CD4^+^ T cells, which are important for the immune surveillance of EBV-infection [79–83].

In addition to the CD4^+^ T cell count, Powls et al. identified an association between the treatment with the non-nucleoside reverse transcriptase inhibitor efavirenz and HL risk, whereas more recent investigations did not identify an association between EFV and HL risk [84–86]. In our study, 6 out of 10 HIV^+^ HL^+^ patients received EFV versus 3 out of 10 of HIV-infected matched controls. However, our sample size did not permit any reasonable statistical analysis.

We did not observe any difference of the overall frequency of TCR γδ T or MAIT cells between HIV-infected patients ± HL. The overall effect of HIV infection on γδ T cells in the PB is in agreement with previous studies, reporting an expansion of TCR Vδ1 cells and a decline of TCR Vδ2 cells, resulting in an inverted Vδ1/Vδ2 ratio [42, 44, 46], and lack of recovery of the altered Vδ1 and Vδ2 distribution upon cART [45]. Similarly, we found a non-reversible reduction of MAIT cells in the PB as previously reported [64].

As cell frequencies and total cell counts alone are not sufficient to determine the involvement of each T cell population in the disease progression, we performed a detailed phenotypic analysis of TCR γδ and MAIT cells in our cohort. The phenotypic analysis comprised immune activation, exhaustion, homing, NK receptor as well as inhibitory markers.

The longitudinal analysis of TCR Vδ1 cells expressing the activation marker CD38 showed an HIV-associated increase similar to what is described for its expression on TCR αβ cells, and in some studies on total TCR γδ cells or on the TCR Vγ9Vδ2 population [87–93]. The increase in the frequency of CD69^+^ MAIT cells was very subtle and in line with earlier observations [63–65, 94]. The frequencies of TCR Vδ1 cells expressing the inhibitory receptors PD-1 and TIM-3 and of MAIT cells expressing PD-1 were overall increased in HIV-infected individuals. Thus, we observed an activated and an exhausted phenotypic profile of these innate immune cells in HIV-infected patients. In fact, HIV-associated T cell activation persists in all kinds of investigated T cell subsets, even in successfully treated HIV-infected patients without detectable viremia, and whether it hampers T cell functions and contributes to T cell immunosenescence remains to be further investigated [95–97].

We also investigated the homing capacities of TCR γδ and MAIT cells in the same patient cohort by studying the expression pattern of CXCR3, CXCR4, CCR5 and CXCR6. The chemokine receptors CXCR3 and CCR5 guide T cells to sites of infection, inflammation and tumors in response to chemokines released by inflammatory tissue and tumor cells [98–100]. CXCR4 is important for homing to the bone marrow [101], and CCR6 is also involved in regulating mucosal immunity, as well as homing of lymphatic cells to the gut mucosal lymphoid tissue [102] and correlates with a Th17-like functional phenotype [55]. TCR γδ cells showed only minor differences between HIV^+^ patients and HC. Instead, MAIT cells expressing CCR5 and CCR6 showed lower frequencies in HIV infection as compared to HCs and their frequencies remained significantly lower even during cART. These results suggest that MAIT cells in HIV-infected individuals might be compromised in their ability to produce IL-17 as well as their homing capacity to specific tissues and sites of inflammation as compared to HCs [55, 103]. We noted a higher frequency of TCR Vδ1 cell and MAIT cells expressing CCR5 and CXCR4, respectively, in HIV^+^ patients w/o HL as compared to HIV^+^ HL^+^ patients just prior to the diagnosis of HL. These findings might point to a particular dysfunction of those cells in the latter group.

We next determined the frequency of cells expressing NK markers and co-stimulatory or inhibitory receptors. A large number of TCR Vδ1 cells expressed CD16 in HIV^+^ w/o HL patients and their frequency was higher in cART treated HIV^+^ w/o HL patients than in HIV^+^ HL^+^ patients. As CD16a is the Fcγ IIIa receptor involved in antibody dependent cytotoxicity and phagocytosis, the observed discrepancy could point to a causal role in the pathogenesis of HL.

TCR Vδ1 cells expressing the inhibitory KIR molecule CD158b also showed a higher frequency in HIV^+^ w/o HL patients compared to HC. Previous studies showed a potent inhibitory effect of KIR molecules on antigen stimulation of TCR γδ cells [104]. Furthermore, in HIV-infected patients an upregulation of KIR expression on CD8^+^ T cells was found, which inhibited their TCR-dependent stimulation [105].

When we performed clustering of high-dimensional FACS data, we found a similar distribution in HIV^+^ w/o HL and HIV^+^ HL^+^ individuals of all TCR γδ populations and MAIT cells. Notably, different clustering was instead observed in healthy controls, thus indicating that the observed differences are driven by differences caused by HIV infection and not HL. The analysis performed with a support vector machine classifier also showed overlapping probability curves for both HIV^+^ w/o HL and HIV^+^ HL^+^ patients. Clear differences were instead observed between HIV-infected and HC and only for the TCR Vδ1 population. Finally, when we analyzed a possible time-dependent effect on phenotypes, we did not see differences between the two groups of HIV^+^-patients, confirming the SVM analysis.

In conclusion, our study provides additional evidence for the ambiguous lower CD4^+^ cell counts just prior to HL as compared to their matched controls even though patients were treated successfully with cART. Our results showed subtle differences between populations of TCR γδ and MAIT cells in HIV^+^-patients with vs. without HL. To what extent these subtle differences contribute to the pathogenesis of HL remains unknown. Future studies need to address their potential role in the development of NADCs in HIV-infected individuals, and whether they might be exploited in novel types of cell therapy.

## Supplementary Information

**Additional file 1.**

## Data Availability

The SHCS (www.shcs.ch) is a prospective cohort study with ongoing enrollment of HIV-infected adults in Switzerland since 1988 [66]. It includes 73% of all diagnosed HIV-infections in Switzerland [67]. Representation has remained stable throughout the study duration. Detailed information on demographics, mode of HIV acquisition, risk behavior, clinical events, co-infections, and treatment is collected using a standard protocol at registration and at intervals of 6 months. Plasma samples are collected every 6–12 months in all study participants.

## Abbreviations

ADCs: AIDS defining cancers
cART: Combined anti-retroviral treatment
HC: Healthy controls
HL: Hodgkin’s lymphoma
MAIT cells: Mucosal-associated invariant T-cells
NADCs: Non-AIDS defining cancers
NK cells: Natural killer cells
PB: Peripheral blood
PBMC: Peripheral blood mononuclear cell
PBS: Phosphate buffered saline
SHCS: Swiss HIV Cohort Study
SVM: Support vector machine
TCR: T-cell receptor
