# Supplementary material for: Immunophenotypic characterization of TCR γδ T cells and MAIT cells in HIV-infected individuals developing Hodgkin’s lymphoma
Source: Infect Agent Cancer. 2021 Apr 17;16:24. doi: 10.1186/s13027-021-00365-4 (PMC8052713; doi:10.1186/s13027-021-00365-4)
Supplement: Supplementary file 1 — Additional file 1. [file 13027_2021_365_MOESM1_ESM.docx]

**Figure S1:**


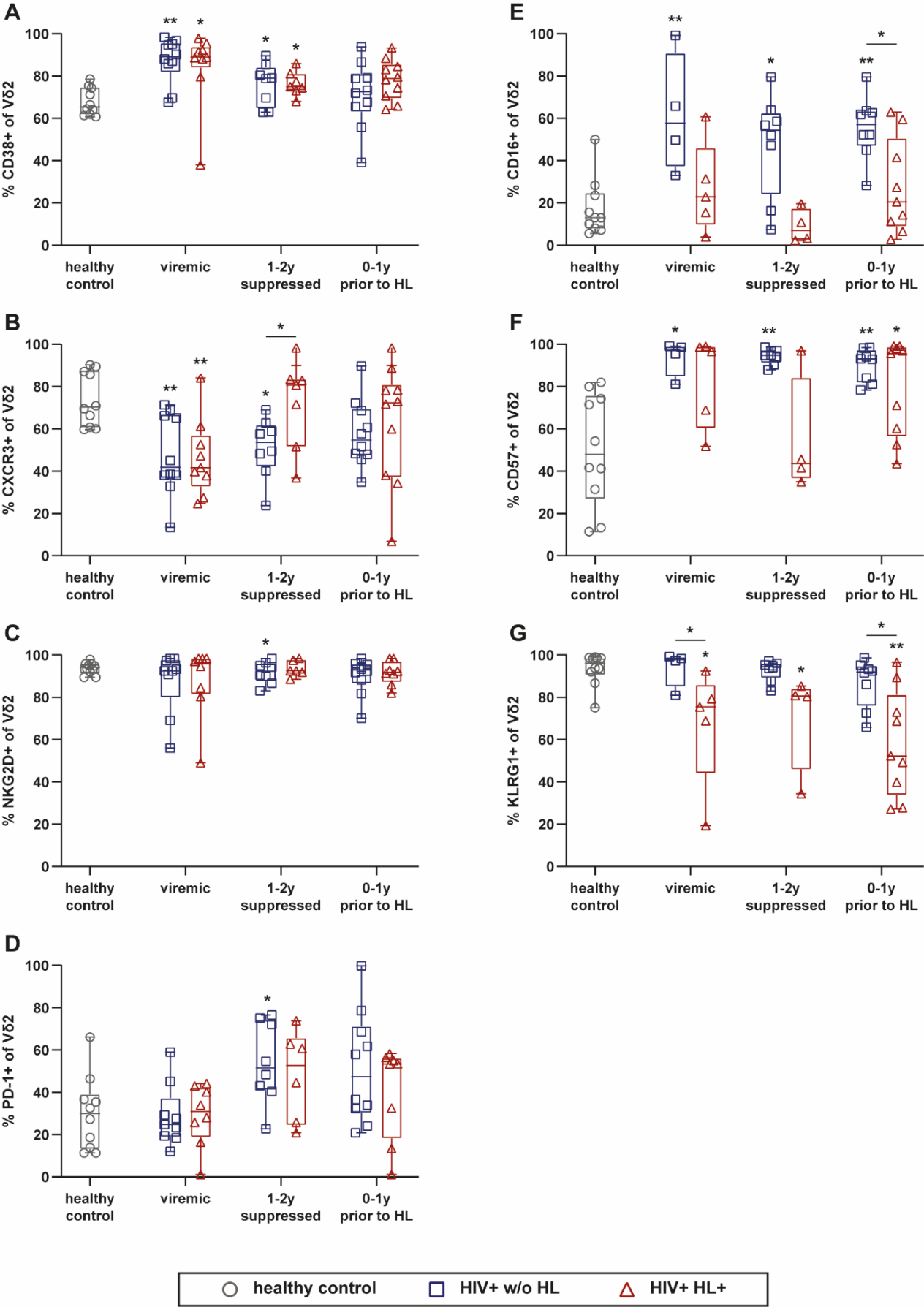


**Supplementary Figure 1: Marker expression on Vδ2.** Whiskers represent minimum and maximum. Analysis of HIV+ HL+ group versus HIV+ control group w/o HL by Wilcoxon signed-rank test, comparison of HIV-infected groups versus HC by Kruskal Wallis with Dunnett’s multiple comparison test. * without indicating line represent significance compared to healthy control. **** p≤0.0001; *** p≤0.001; ** p≤0.01; *p≤0.05. (PB) peripheral blood, (HL) Hodgkin’s lymphoma.

**Figure S2:**


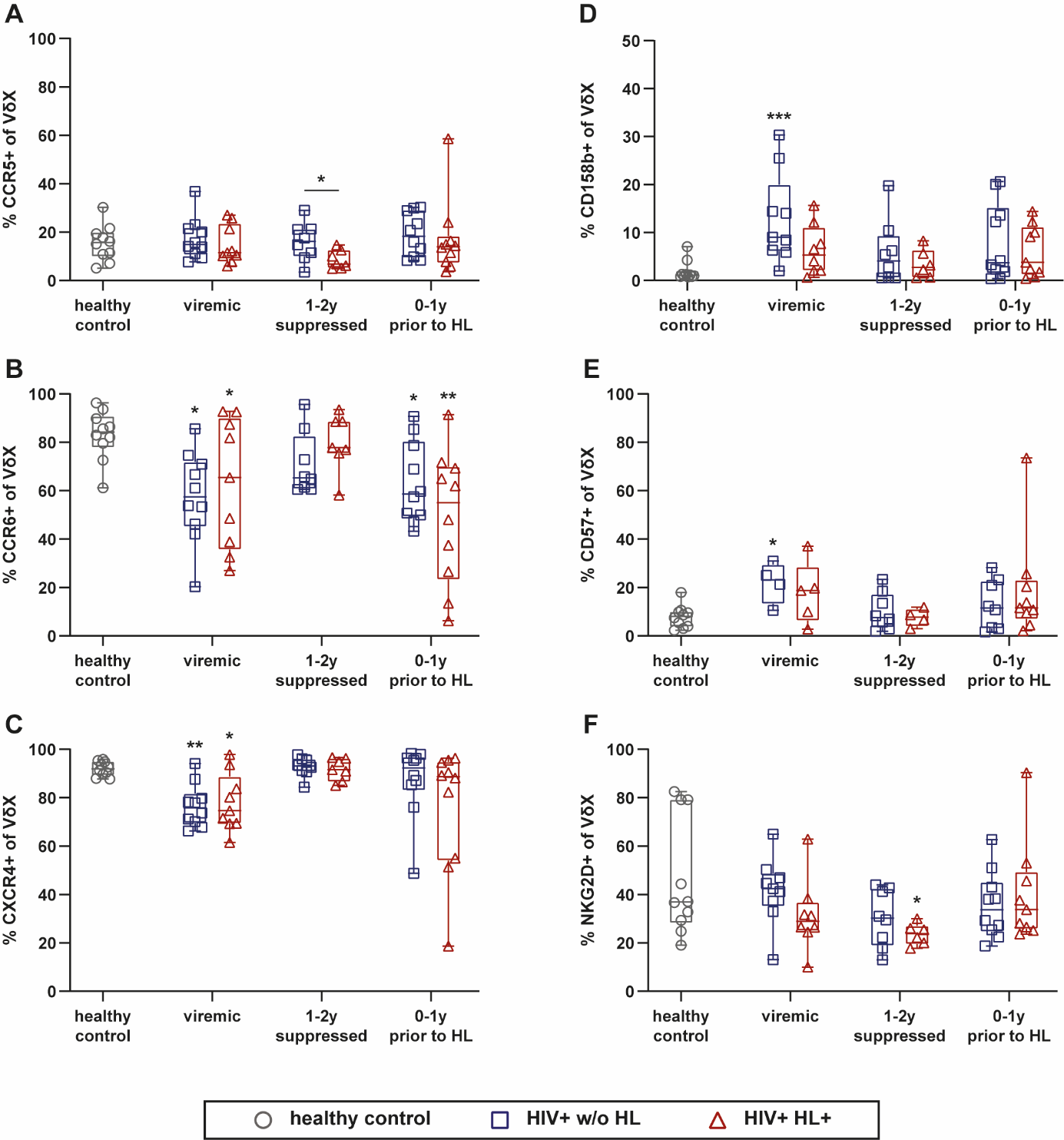


**Supplementary Figure 2: Marker expression on VδX.** Whiskers represent minimum and maximum. Analysis of HIV+ HL+ group versus HIV+ control group w/o HL by Wilcoxon signed-rank test, comparison of HIV-infected groups versus HC by Kruskal Wallis with Dunnett’s multiple comparison test. * without indicating line represent significance compared to healthy control. **** p≤0.0001; *** p≤0.001; ** p≤0.01; *p≤0.05. (PB) peripheral blood, (HL) Hodgkin’s lymphoma.

### Figure S3:


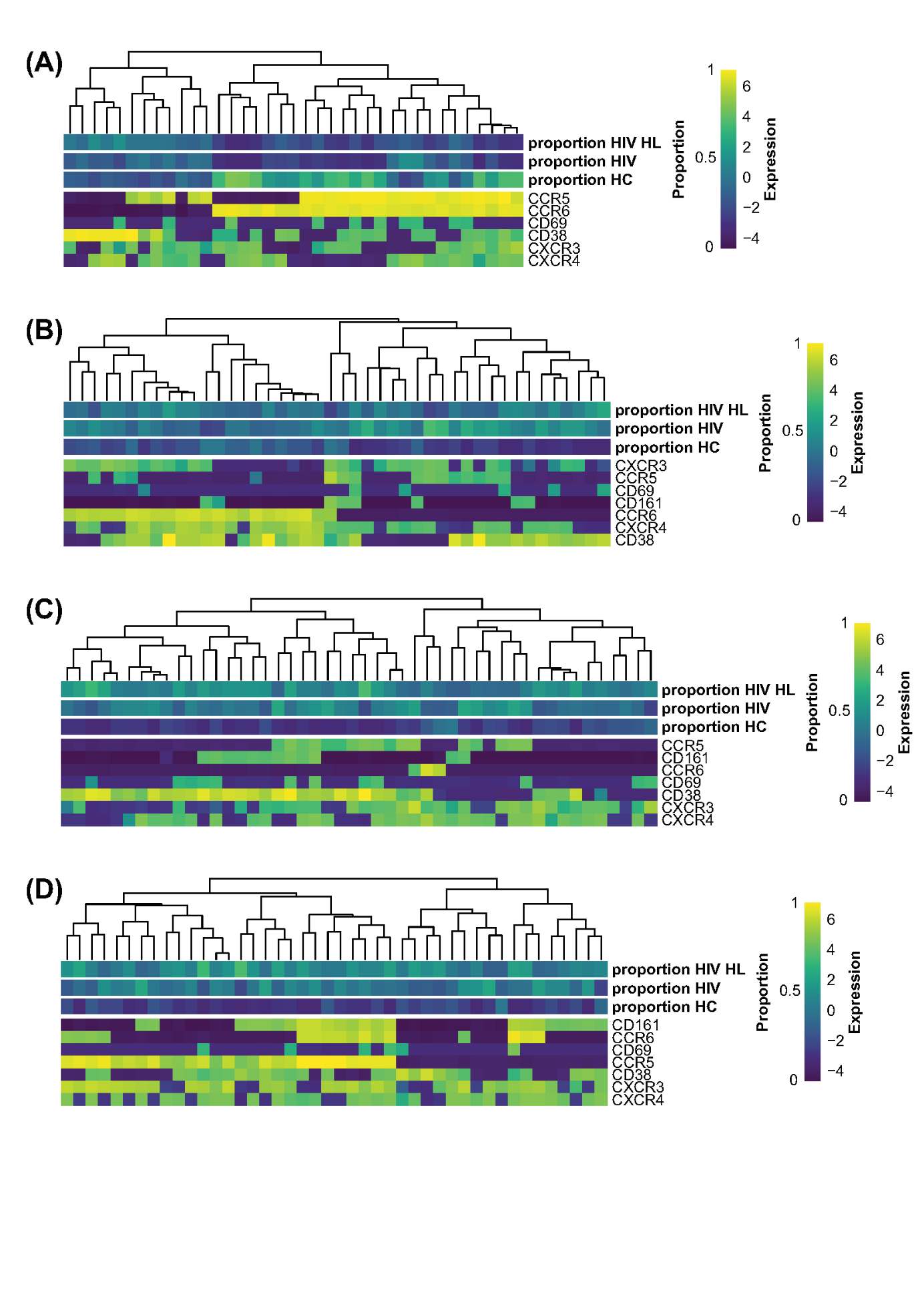


### Supplementary Figure 3: Expression of chemokine receptors in PhenoGraph clusters. Median expression of chemokine receptor markers in clusters identified by PhenoGraph in (A) MAIT, Gamma-delta (B) Vδx, (C) Vδ1 and (D) Vδ2 populations. The proportion of cells from HC, HIV and HIV HL patients within each cluster is represented in the top three rows of the heatmap.

### Figure S4:


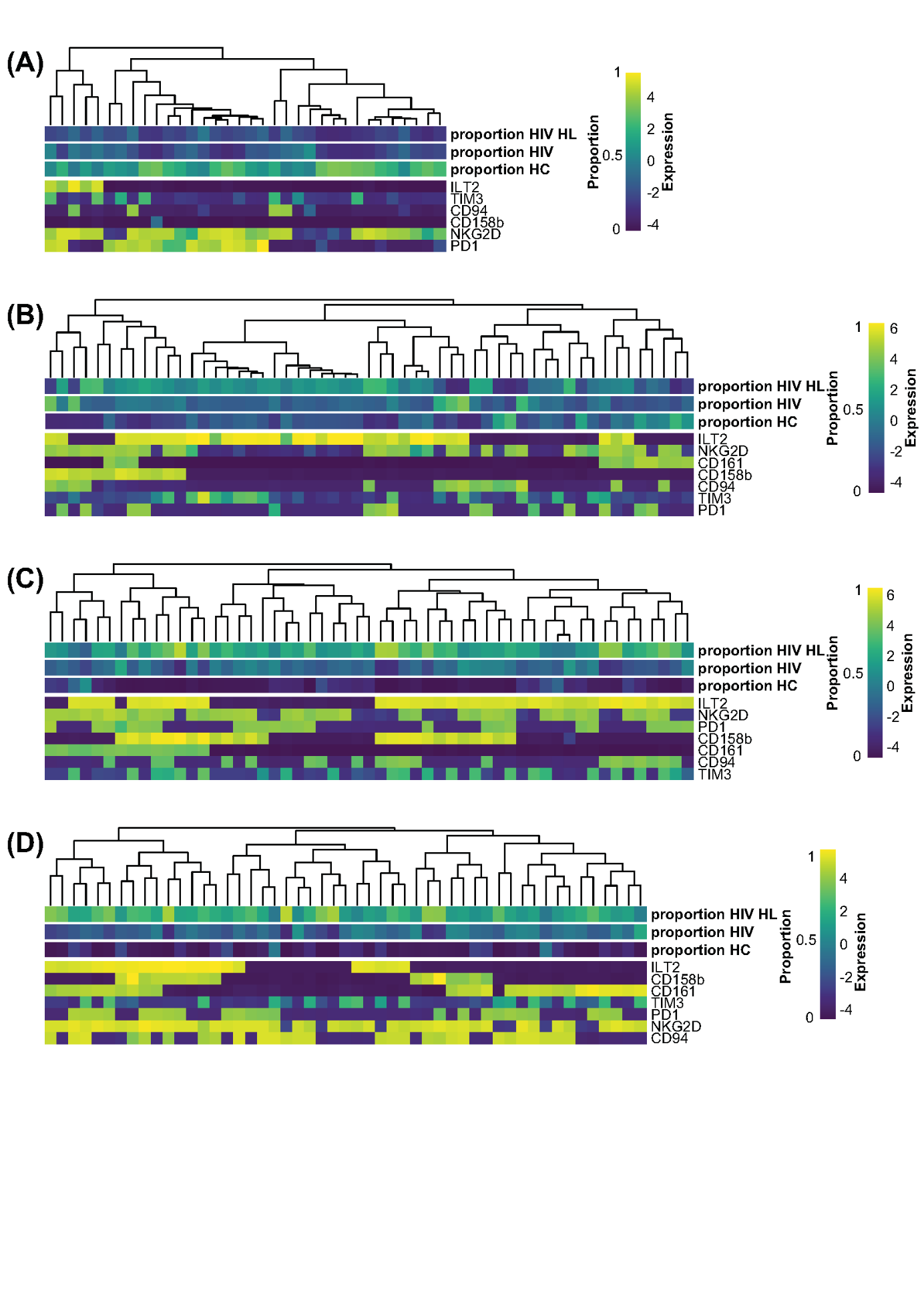


### Supplementary Figure 4: Expression of exhaustion markers in PhenoGraph clusters. Median expression of exhaustion markers in clusters identified by PhenoGraph in (A) MAIT, Gamma-delta (B) Vδx, (C) Vδ1 and (D) Vδ2 populations. The proportion of cells from HC, HIV and HIV HL patients within each cluster is represented in the top three rows of the heatmap.

### Figure S5:

###
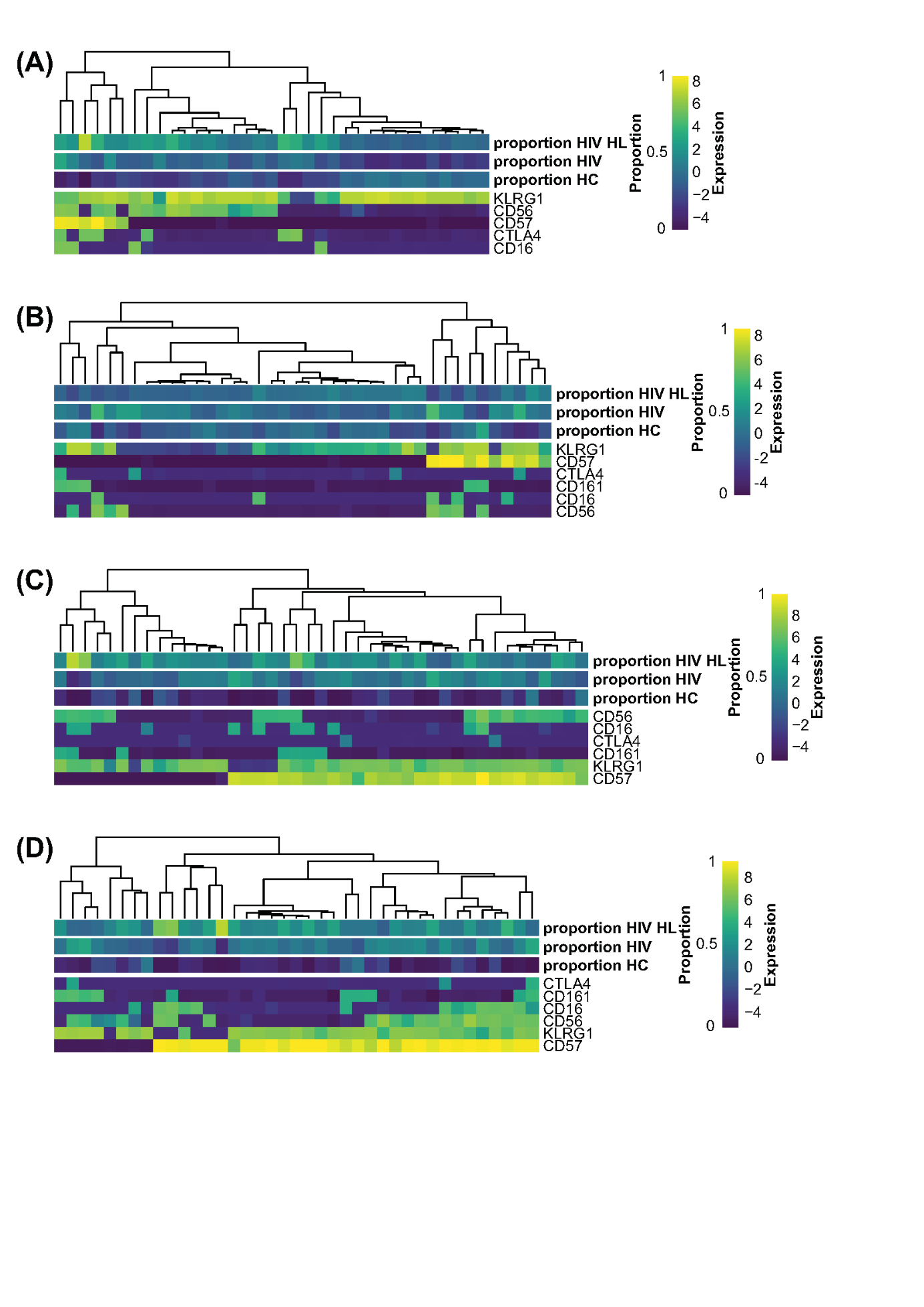


### Supplementary Figure 5: Expression of NK markers in PhenoGraph clusters. Median expression of NK markers in clusters identified by PhenoGraph in (A) MAIT, Gamma-delta (B) Vδx, (C) Vδ1 and (D) Vδ2 populations. The proportion of cells from HC, HIV and HIV HL patients within each cluster is represented in the top three rows of the heatmap.

### Figure S6:


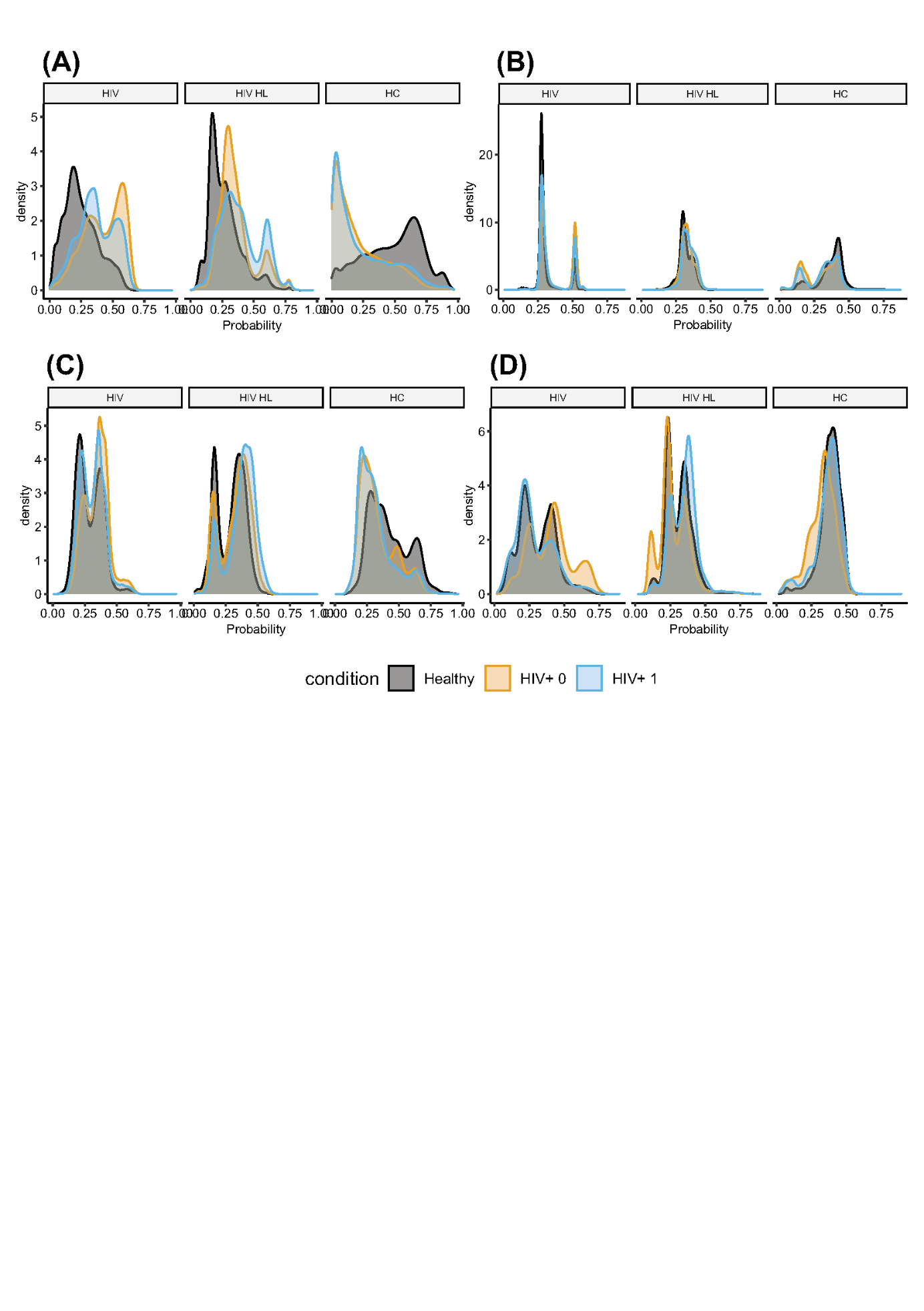


**Supplementary** **Figure 6: Classification of cells by independent SVM classifier models trained on chemokine receptor expression.** Separate SVM models were trained on 20% of each population, evenly sampling over patients, conditions and time points, each model was then used to classify all cells as either HC, HIV or HIV HL. The models’ condition membership probability for cells in (A) MAIT, Gamma-delta (B) Vδx, (C) Vδ1 and (D) Vδ2 populations to be classified as coming from HC, HIV or HIV HL patients is shown.

### Figure S7:

**
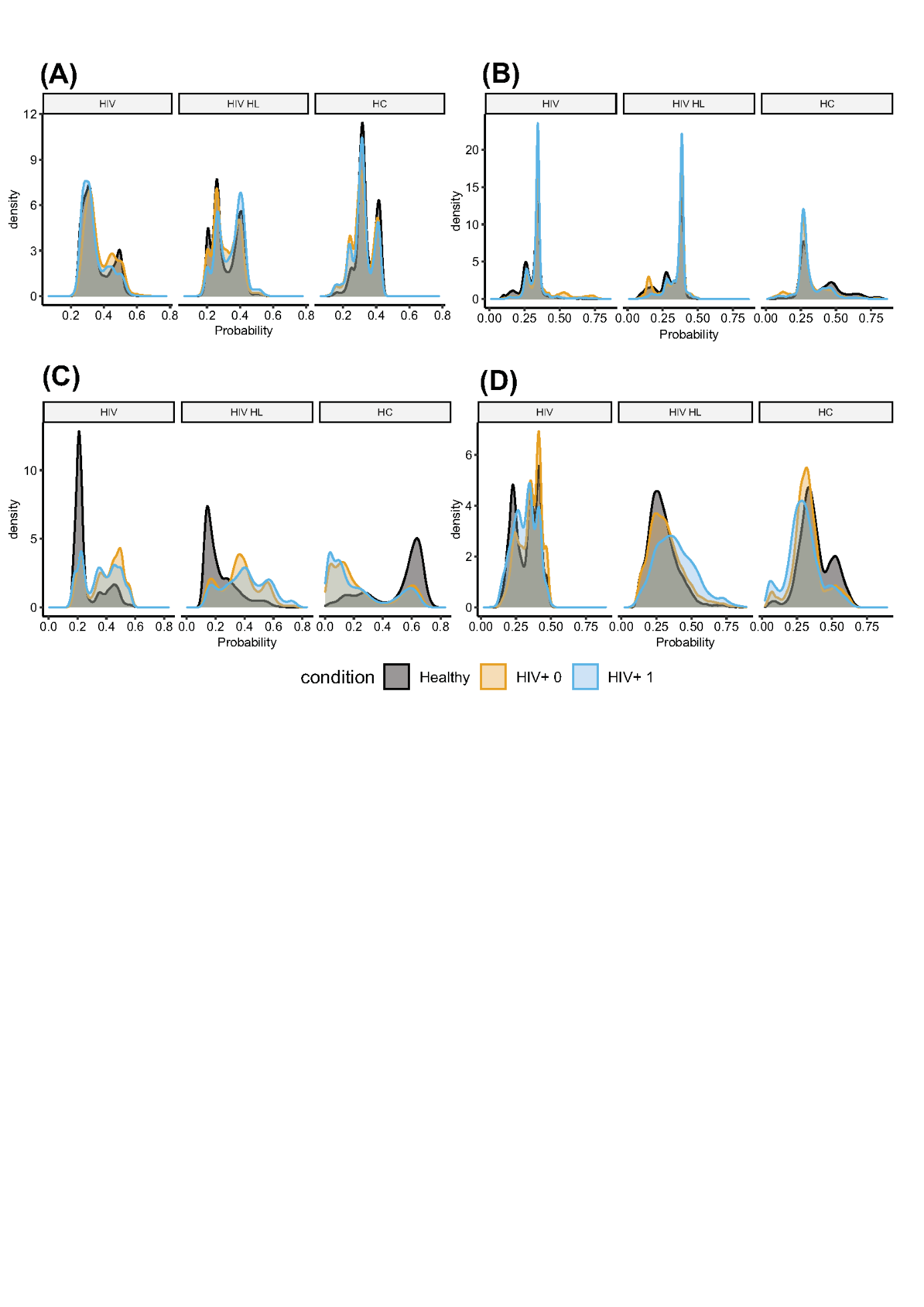
**

**Supplementary** **Figure 7: Classification of cells by independent SVM classifier models trained on exhaustion marker expression.** Separate SVM models were trained on 20% of each population, evenly sampling over patients, conditions and time points, each model was then used to classify all cells as either HC, HIV or HIV HL. The models’ condition membership probability for cells in (A) MAIT, Gamma-delta (B) Vδx, (C) Vδ1 and (D) Vδ2 populations to be classified as coming from HC, HIV or HIV HL patients is shown.

### Figure S8:


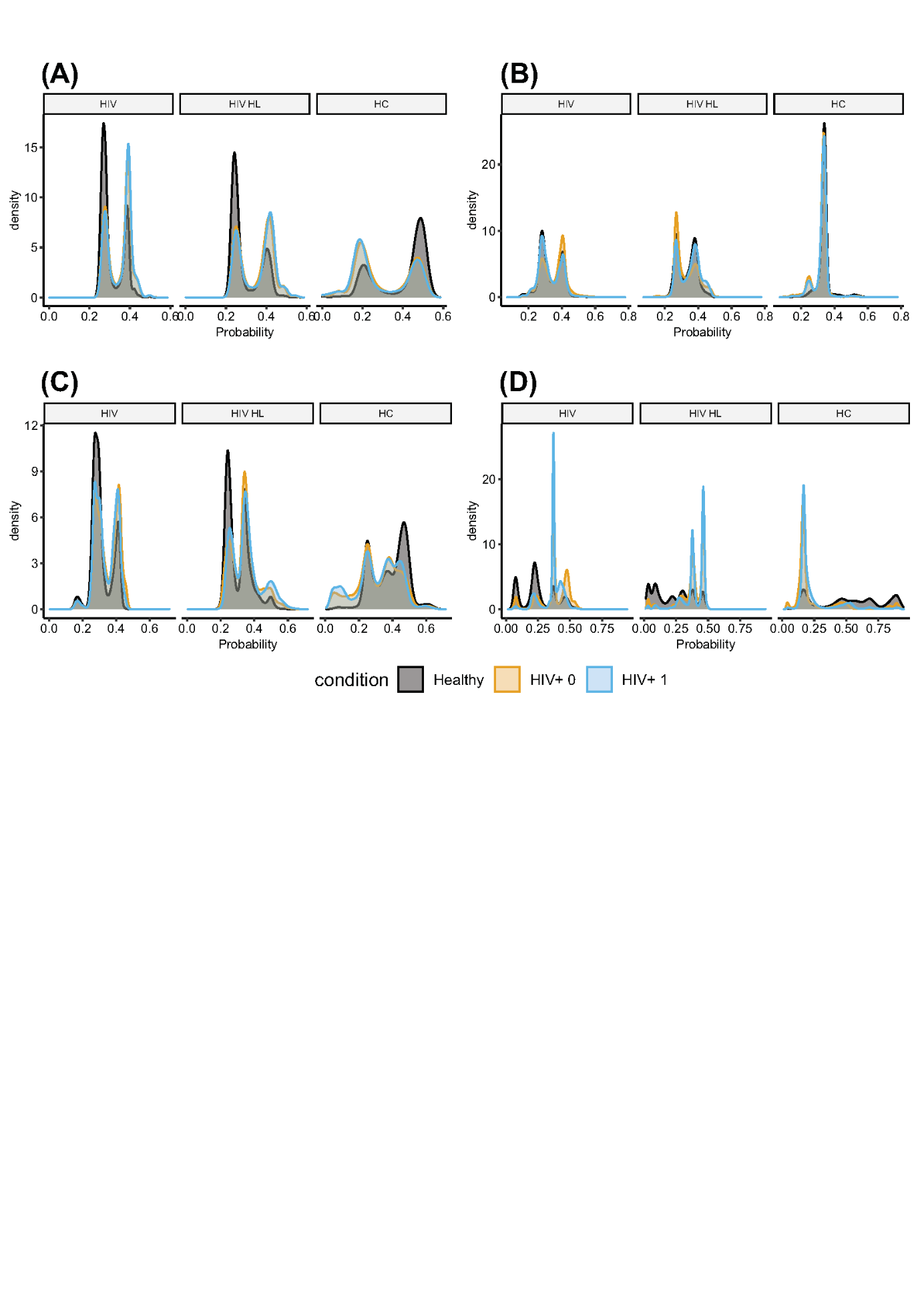


**Supplementary** **Figure 8: Classification of cells by independent SVM classifier models trained on NK marker expression.** Separate SVM models were trained on 20% of each population, evenly sampling over patients, conditions and time points, each model was then used to classify all cells as either HC, HIV or HIV HL. The models’ condition membership probability for cells in (A) MAIT, Gamma-delta (B) Vδx, (C) Vδ1 and (D) Vδ2 populations to be classified as coming from HC, HIV or HIV HL patients is shown.

### Figure S9:

**
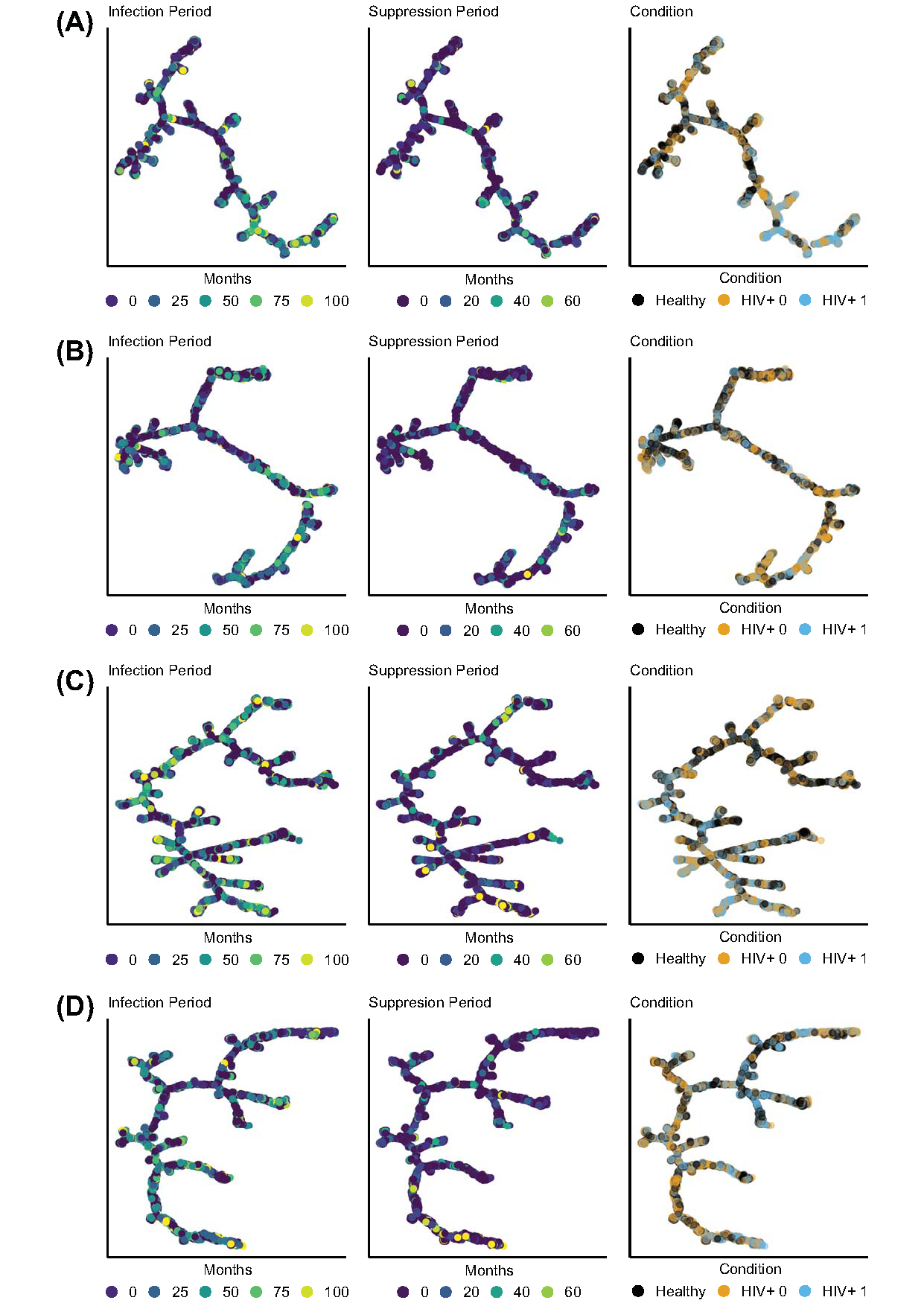
**

**Supplementary** **Figure 9: Inference of Time-dependent Changes in Chemokine Receptor Expression.** Data for each population, down-sampled evenly over patients, conditions and time-points to 1e4 cells, was used to predict phenotype trajectories using DDRTree. Predicted trajectories show distribution of time-dependent variables (infection and treatment duration) and cells from HC, HIV and HIV HL patients in response to chemokine receptor phenotypes over; (A) MAIT, Gamma-delta (B) Vδx, (C) Vδ1 and (D) Vδ2 populations.

### Figure S10:


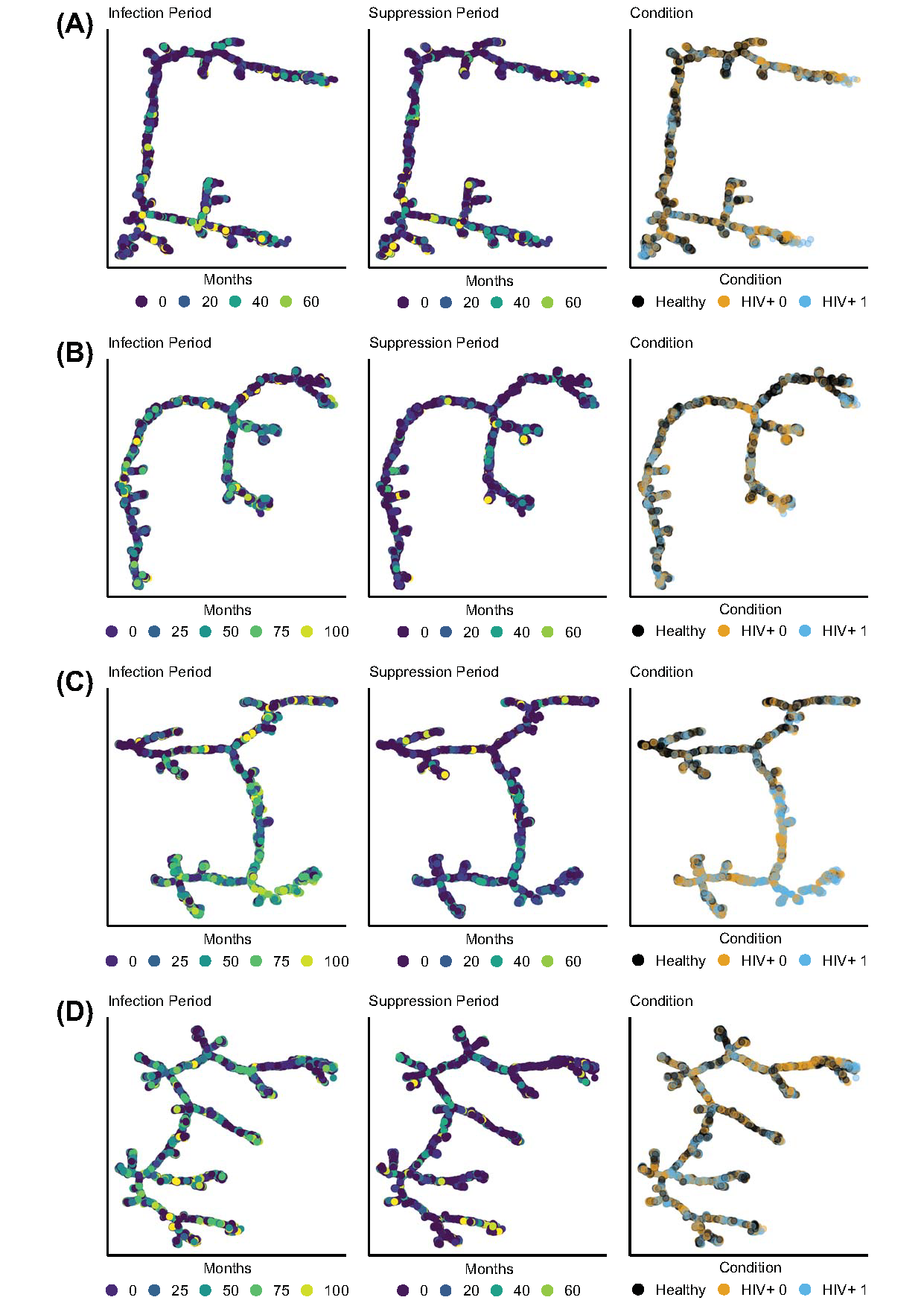


**Supplementary** **Figure 10: Inference of Time-dependent Changes in Exhaustion Phenotypes.** Data for each population, down-sampled evenly over patients, conditions and time-points to 1e4 cells, was used to predict phenotype trajectories using DDRTree. Predicted trajectories show distribution of time-dependent variables (infection and treatment duration) and cells from HC, HIV and HIV HL patients in response to exhaustion marker phenotypes over; (A) MAIT, Gamma-delta (B) Vδx, (C) Vδ1 and (D) Vδ2 populations.

### Figure S11:

###
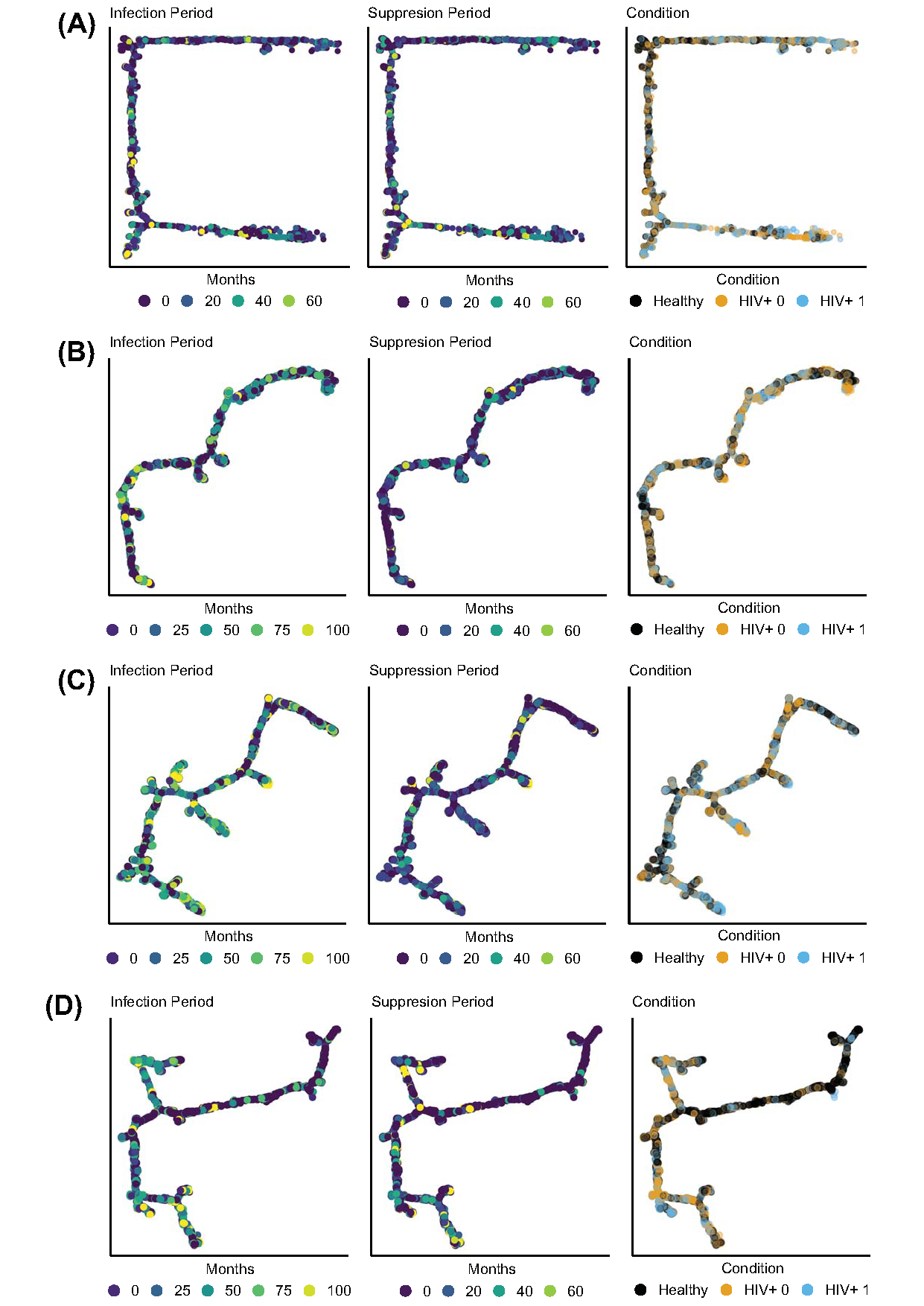


### Supplementary Figure 11: Inference of Time-dependent Changes in NK-like Phenotypes. Data for each population, down-sampled evenly over patients, conditions and time-points to 1e4 cells, was used to predict phenotype trajectories using DDRTree. Predicted trajectories show distribution of time-dependent variables (infection and treatment duration) and cells from HC, HIV and HIV HL patients in response to NK marker phenotypes over; (A) MAIT, Gamma-delta (B) Vδx, (C) Vδ1 and (D) Vδ2 populations.
